# Increased risk of ischemic stroke in patients with burn injury: a nationwide cohort study in Taiwan

**DOI:** 10.1186/s13049-016-0236-1

**Published:** 2016-04-06

**Authors:** Tzu-Yao Hung, Yi-Kung Lee, Ming-Yuan Huang, Chen-Yang Hsu, Yung-Cheng Su

**Affiliations:** Emergency Department, Zhongxing Branch of Taipei City Hospital, Taipei, Taiwan; School of Medicine, Tzu Chi University, Hualien, Taiwan; Emergency Department, Dalin Tzu Chi Hospital, Buddhist Tzu Chi Medical Foundation, No. 2, Minsheng Rd., Dalin Township, Chiayi County 622, Taiwan R.O.C; Department of Emergency Medicine, Mackay Memorial Hospital, Taipei, Taiwan; Department of Public Heath, National Taiwan University, Taipei, Taiwan

**Keywords:** Burns, Ischemic stroke, Cohort studies

## Abstract

**Background:**

Conflicting results have been obtained by studies attempting to assess the risks of ischemic stroke in patients with burn injury, while the long-term risk of stroke in survivors of burn injury remains unexplored. We evaluated whether the risk of ischemic stroke in patients hospitalized with burn injury in Taiwan is higher when compared to the general population.

**Methods:**

The data from one million National Health Insurance (NHI) adult beneficiaries were evaluated from January 1, 2005 until December 31, 2012 to identify those who developed ischemic stroke. Each identified patient with burn injury was matched with one hundred unexposed patients based on a high-dimensional propensity score. Cox regression models were applied to compare the risks of the development of ischemic stroke in the matched cohorts.

**Results:**

A total of 743,237 patients were enrolled. After matching, 1,763 burn injury patients and 176,300 unexposed patients were selected and compared. The adjusted hazard ratio of ischemic stroke was significantly increased in burn injury patients (1.84; 95 % CI, 1.43–2.36). A subgroup analysis based on patients who survived longer than 12 months in the matched cohort also revealed higher hazard ratio in the burn injury patients (1.54; 95 % CI, 1.11–2.13).

**Conclusion:**

The risk of ischemic stroke is significantly higher in patients hospitalized with burn injury than in the general population, and these risks may extend longer than expected.

## Background

Stroke remains one of the leading causes of long-term disability and mortality, with a worldwide incidence of 4.2–6.5 per 1000 person-years for people aged 55 or older, resulting in a significant societal economic burden [1–3]. Apart from patient management, early identification of risk factors for the development of stroke is critical to reduce morbidity and mortality. Several precipitating co-morbidity factors have been reported to be associated with stroke onset, including hypertension, diabetes, hyperlipidemia, atrial fibrillation, and a history of smoking [4]. Recently, the potential relationship between burn injury and stroke has begun to receive attention from the clinical community.

It is well known that cerebral complications are common in victims of burn injury [5, 6]. Hypovolemia, during the acute stage of burn injury, may induce poor cerebral perfusion, resulting in ischemic stroke. In addition, sepsis, a common complication in burn patients, may represent another risk factor of stroke [7], while a state of acquired hypercoagulopathy that is often encountered following burn injury may present a further risk of stroke in these patients [8, 9].

To our knowledge, few studies exist concerning the incidence of ischemic stroke after burn injury, and the results are contradictory [6, 10]. Moreover, the long-term risk of stroke in survivors of burn injury remains unexplored. In the present study, a large administrative database was used to elucidate the risks of ischemic stroke in burn injury patients in Taiwan. High-dimensional propensity score (hdPS) matching (i.e., a semi-automated statistical method) was used to address possible confounding. The results of this study may help clinicians to identify individuals at possible risk for stroke.

## Methods

### Ethics statement

This study was conducted in accordance to the tenets of the Helsinki Declaration. The Institutional Review Board of Dalin Tzu Chi Hospital, Buddhist Tzu Chi Medical Foundation, Taiwan, approved the study. The need for informed consent was waived because patient record/information was anonymous and no identifiers were employed.

### Database

The Taiwanese National Health Insurance (NHI) program was initiated in 1995, and has been described in detail previously [11–13]. Utilizing the Longitudinal Health Insurance Database (2005), a dataset comprising 1,000,000 individuals who were representative beneficiaries of the NHI program was randomly sampled.

### Study population

**Fig. 1:**
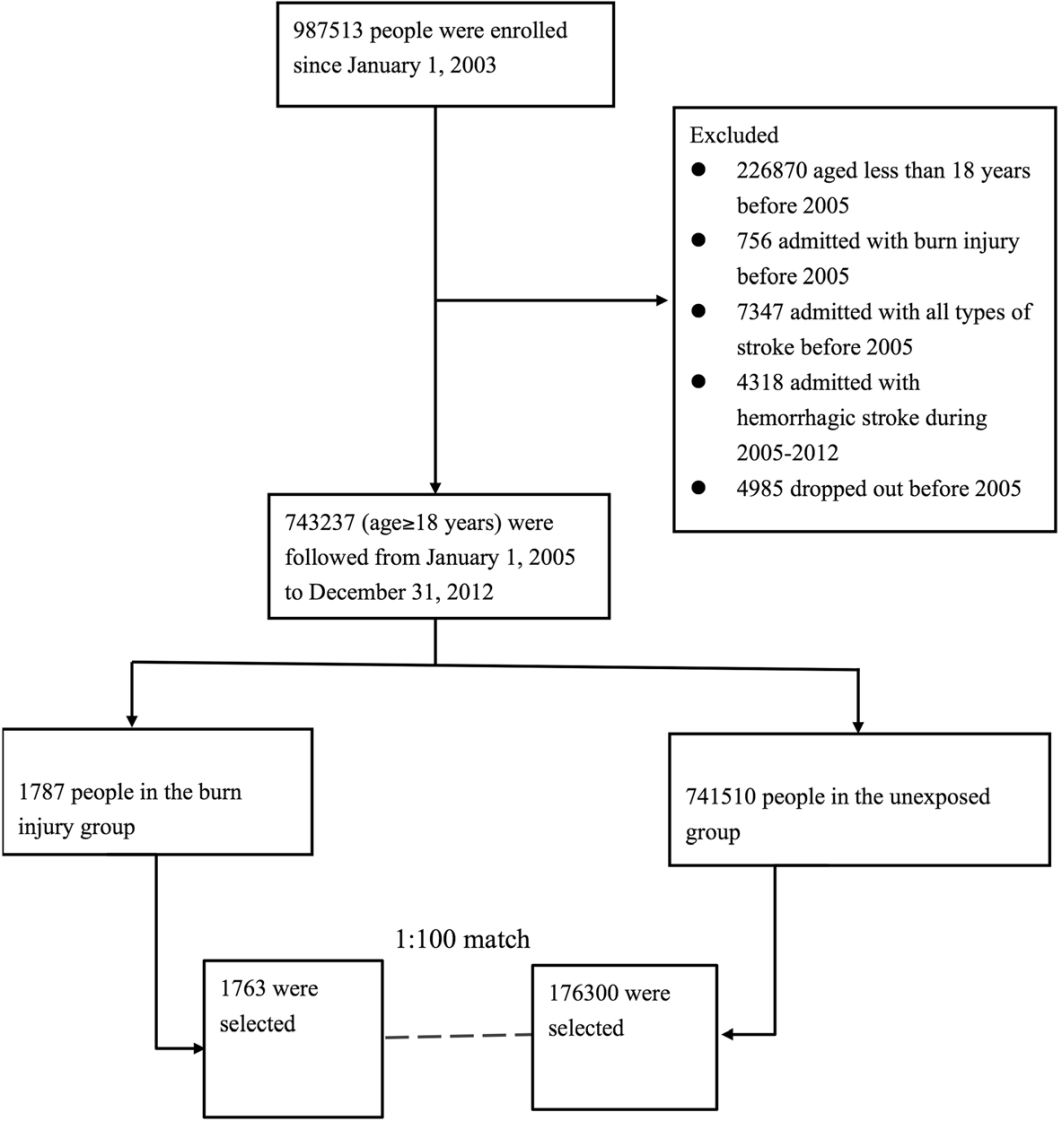
Flow diagram of the population-based study

The representative population sample was systematically tracked between January 1, 2003 and December 31, 2012. Individuals aged >18 years who were alive in 2005 were initially identified. Burn injury was defined by the ICD-9-CM codes 940–949. Only patients hospitalized for burn injuries were enrolled in the study, thereby excluding those sustaining mild burns. Ischemic stroke was defined by ICD-9-CM codes 433–437 [14]. To maximize the scope of our study, we included only those patients hospitalized for stroke. Patients with burn injuries and any type of stroke diagnosed before January 1, 2005 were excluded from the study to ensure all enrolled cases and events were new. We chose to focus on ischemic stroke alone to avoid any possible overlap between the ICD-9-CM codes for traumatic intracranial hemorrhage and hemorrhagic stroke. Patients diagnosed with hemorrhagic stroke during follow-up were also excluded. After excluding patients according to the aforementioned criteria, we identified 1,787 patients hospitalized with burn injuries and 741,510 without burn injuries. Each group was followed from the hospitalization date or January 1, 2005 (baseline) to December 31, 2012 (end of the study period) for verification of a diagnosis of ischemic stroke during the study period. Patients who were no longer beneficiaries of the NHI Program (due to death or transfer) or were still healthy at the end of the follow-up period were carefully monitored (Fig. 1).

### Validation of exposure and outcome

We investigated the validity of the ICD-9-CM codes for the identification of burn injuries and ischemic stroke. The codes were validated by analyzing medical records randomly selected from the electronic database from 2010 to 2012 at the Dalin Tzu Chi Hospital, Buddhist Tzu Chi Medical Foundation. We then estimated the positive predictive value (PPV). A systematic chart review confirmed the diagnosis in 45 of 50 randomly selected patients with codes for burn injuries, thereby validating a PPV of 90 % (95 % CI, 78.2 %–96.7 %). In addition, the diagnosis was also confirmed in 47 of 50 randomly selected patients with codes for ischemic stroke, validating a PPV of 94 % (95 % CI, 83.5 %–98.7 %).

### Pre-specified covariates

To better understand the effect of burn injury on the risk of ischemic stroke, several covariates were selected including age, sex, urbanization level (i.e., urban, suburban, and rural), and socioeconomic status (SES). Income-related insurance payment amounts were used as a proxy measure of individual SES at follow-up [15]. The prevalence of selected comorbidity factors (i.e., diabetes, hypertension, coronary artery disease, hyperlipidemia, history of alcohol intoxication, malignancies, heart failure, atrial fibrillation, history of smoking and peripheral artery disease) and the Charlson Comorbidity Index (CCI) were determined using discharge diagnoses either during outpatient clinic visits or hospitalizations before January 1, 2005. The CCI represents a scoring system with weighted factors of important concomitant diseases and is widely used in the ICD-9-CM coded administrative databases [14].

### High dimensional propensity scores

The high-dimensional propensity score (hdPS), available as an SAS macro from the Brigham and Women’s Hospital [16, 17], is a multistep, empirically driven algorithm that is used to adjust for confounding bias. Candidate covariates from predefined data dimensions, such as clinical treatment received, medications administered based on the Anatomical Therapeutic Chemical (ATC) Classification System, and coded diagnoses reported in this study from January 1, 2003 to December 31, 2004, were identified using the SAS macro. It automatically assesses the repetition of the same codes, prioritizes covariates, and identifies covariates for adjustment. We selected the top 500 variables most likely to result in a bias.

In addition to these 500 variables, the pre-selected covariates were analyzed with a logistic regression model to estimate the predicted probability (propensity score) of exposure of the study subjects to burn injuries when compared with the healthy population. This exposure is conditional on all the included covariates. After using a standard greedy matching algorithm to match all burn injury patients with 100 non-burn injury patients having the closest propensity scores [18, 19], we compared the risk for the development of ischemic stroke between the two groups.

### Statistical analysis

We used an SAS statistical package (Version 9.4, SAS Institute, Inc., Cary, NC, USA), and STATA (Version 11.2, StataCorp, College Station, TX, USA) for all data analyses. All covariates except age and propensity scores were included as categorical variables (age and propensity scores were considered continuous variables). Categorical variables and continuous variables were compared using Pearson’s chi-square test and a *t* test, respectively, to determine the baseline heterogeneity within the two groups. The cumulative risks of ischemic stroke were first determined by plotting the Nelson–Aalen curves. The hazard ratios (HRs) for ischemic stroke in patients with burn injury in the matched group were calculated using the Cox proportional hazard regression models after adjustment for age, gender, urbanization level, SES, diabetes, hypertension, coronary artery disease, hyperlipidemia, malignancies, heart failure, atrial fibrillation, peripheral artery disease, history of alcohol use and smoking, and CCI. Adjusted HRs were analyzed on the basis of the following parameters: (1) Hospitalization periods for subjects with or without burn injuries from baseline through the end of the study and (2) 12-month periods after hospitalization for burn injury and non-burn injury subjects from baseline through the end of the study to evaluate the long-term impact of burn injuries on the risk of ischemic stroke onset.

To further validate the accuracy of our results, we also performed sensitivity analyses to evaluate the extent of the effect of an unmeasured confounder on the results [20]. A two-tailed *p*-value of 0.05 was considered significant.

## Results

**Fig. 2:**
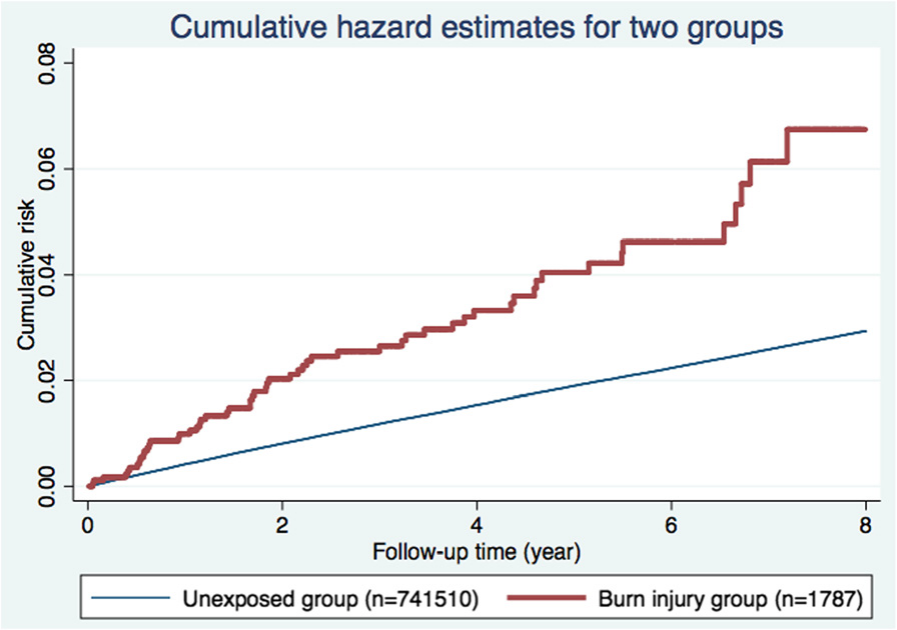
Nelson-Aalen curves showing a higher cumulative risk of ischemic stroke in the burn injury group

The distribution of demographic characteristics and selected morbidities in both study groups is shown in Table 1. There were 1,787 patients in the burn injury group and 741,510 in the unexposed group. The total follow-up time was 6,564 and 5,581,503 person-years, and the mean follow-up period was 3.67 and 7.53 years, respectively. Patients with burn injury were mostly male, older, and more likely to have diabetes, hypertension, coronary artery disease, a history of alcohol intoxication, heart failure, and higher CCI. By the end of the follow-up period, 20,562 patients had been admitted for ischemic stroke (66 patients in the burn injury group and 20,496 in the unexposed group). The crude hazard ratio (HR) of ischemic stroke for the burn injury group was 2.64 (95 % confidence interval [CI], 2.07–3.36), and the Nelson-Aalen plot showed a higher cumulative risk in the burn injury group (Fig. 2).

**Table 1:** Baseline characteristics of the burn injury group and the unexposed group

Variables, n (%)	Burn injury group		Unexposed group		*P-*value
	*n* = 1787		*n* = 741510		
Male	1042	58.3	362518	48.9	**<0.001**
Mean age (SD)	46.6	17.4	42.6	16.4	**<0.001**
Socioeconomic status					**<0.001**
Low	867	48.5	312208	42.1	
Moderate	740	41.4	292075	39.4	
High	180	10.1	137167	18.5	
Urbanization level					**<0.001**
Urban	867	48.5	221749	29.9	
Suburban	740	41.4	341946	46.1	
Rural	180	10.1	177755	24.0	
Charlson Comorbidity Index					**<0.001**
0	1099	61.5	501427	67.6	
1	412	23.1	147724	19.9	
≥2	276	15.4	92299	12.4	
Diabetes	191	10.7	50273	6.8	**<0.001**
Hypertension	298	16.7	106683	14.4	**0.006**
Coronary artery disease	140	7.8	42221	5.7	**<0.001**
Hyperlipidemia	176	9.9	64947	8.8	0.104
History of alcohol intoxication	40	2.2	5288	0.7	**<0.001**
Malignancies	23	1.3	11861	1.6	0.293
heart failure	20	1.1	4402	0.6	**0.004**
Atrial fibrillation	6	0.3	2430	0.3	0.953
Smoking	1	0.1	318	0.04	0.790
Peripheral artery disease	13	0.7	3898	0.5	0.239
Stroke	66	3.7	20496	2.8	**0.017**
Mean Propensity score (SD)	0.0069	0.0219	0.0024	0.0031	**<0.001**

p-Values less than 0.05 are presented as bold type

Next, we matched 1,763 patients in the burn injury group with 176,300 in the unexposed group. In the subcohort, 6,860 patients were admitted for ischemic stroke, (62 in the burn injury group and 6,798 in the unexposed group. The distribution of all pre-specified covariates within the two subgroups was found to be similar and summarized in Table 2. A multivariate Cox regression model based on the pre-specified covariates was used to evaluate the adjusted HRs of ischemic stroke, which after controlling for age, gender, urbanization level, SES, diabetes, hypertension, coronary artery disease, hyperlipidemia, history of alcohol use, malignancies, heart failure, atrial fibrillation, history of smoking, peripheral artery disease and CCI, remained significantly higher in patients with burn injury (1.84; 95 % CI, 1.43–2.36; *p* < 0.001). All statistical results are summarized in Table 3.

**Table 2:** Baseline characteristics of the propensity matched cohort

Variables, n(%)	Burn injury group		Unexposed group		*P-*value
	*n* = 1763		*n* = 176300		
Male	1028	58.3	101561	57.6	0.552
Mean age (SD)	46.6	17.4	46.0	17.3	0.155
Socioeconomic status					0.410
Low	847	48.0	82936	47.0	
Moderate	736	41.8	73690	41.8	
High	180	10.2	19674	11.2	
Urbanization level					0.546
Urban	365	20.7	38300	21.7	
Suburban	834	47.3	82999	47.1	
Rural	564	32.0	55001	31.2	
Charlson Comorbidity Index					0.441
0	1096	62.2	111944	63.5	
1	402	22.8	39449	22.4	
≥2	265	15.0	24907	14.1	
Diabetes	181	10.3	16578	9.4	0.217
Hypertension	291	16.5	28058	15.9	0.500
Coronary artery disease	135	7.7	12299	7.0	0.264
Hyperlipidemia	173	9.8	16819	9.5	0.698
History of alcohol intoxication	34	1.9	2653	1.5	0.147
Malignancies	23	1.3	2300	1.3	1.000
heart failure	20	1.1	1630	0.9	0.360
Atrial fibrillation	6	0.3	555	0.3	0.849
Smoking	1	0.1	81	0.1	0.834
Peripheral artery disease	13	0.7	1169	0.7	0.702
Stroke	62	3.5	6798	3.9	0.462
Mean Propensity score (SD)	0.0051	0.0063	0.0041	0.0043	**<0.001**

p-Values less than 0.05 are presented as bold type

**Table 3:** Adjusted HRs for patients with burn injury in the propensity score matched cohort

Variables	Hazard ratio	95 % confidence interval	*P-*value
Burn injury	1.84	1.43–2.36	**<0.001**
Male	1.39	1.32–1.46	**<0.001**
Patient age	1.07	1.06–1.07	**<0.001**
Socioeconomic status			
Low	1	--	--
Moderate	0.68	0.65–0.72	**<0.001**
High	0.42	0.35–0.50	**<0.001**
Urbanization level			
Urban	1	--	--
Suburban	1.12	1.05–1.20	**<0.001**
Rural	1.38	1.28–1.48	**<0.001**
Charlson Comorbidity Index			
0	1	--	--
1	1.31	1.23–1.41	**<0.001**
≥2	1.65	1.54–1.77	**<0.001**
Diabetes	1.76	1.66–1.87	**<0.001**
Hypertension	1.72	1.62–1.81	**<0.001**
Coronary artery disease	0.99	0.94–1.06	0.946
Hyperlipidemia	0.94	0.88–0.99	0.043
History of alcohol intoxication	1.49	1.25–1.79	**<0.001**
Malignancies	0.86	0.74–0.99	0.045
Heart failure	1.15	1.02–1.30	0.028
Atrial fibrillation	1.27	1.04–1.54	0.017
Smoking	0.98	0.37–2.61	0.963
Peripheral artery disease	1.07	0.91–1.26	0.417

p-Values less than 0.05 are presented as bold type

A subgroup analysis based on subjects who were followed longer than 12 months in the matched cohort was performed (1,461 patients in the burn injury group and 172,543 in the unexposed group). The adjusted HR for ischemic stroke remained statistically significant. (1.54; 95 % CI, 1.11—2.13) (Table 4).

**Table 4:** Adjusted HRs for patients followed longer than 12 months in the matched cohort

Variables	Hazard ratio	95 % confidence interval	*P-*value
Burn injury	1.54	1.11–2.13	**0.009**
Male	1.39	1.31–1.46	**<0.001**
Patient age	1.07	1.06–1.07	**<0.001**
Socioeconomic status			
Low	1	--	--
Moderate	0.71	0.67–0.76	**<0.001**
High	0.42	0.35–0.51	**<0.001**
Urbanization level			
Urban	1	--	--
Suburban	1.12	1.04–1.21	**0.003**
Rural	1.37	1.27–1.48	**<0.001**
Charlson Comorbidity Index			
0	1	--	--
1	1.31	1.22–1.41	**<0.001**
≥2	1.57	1.45–1.69	**<0.001**
Diabetes	1.76	1.65–1.87	**<0.001**
Hypertension	1.65	1.55–1.75	**<0.001**
Coronary artery disease	0.97	0.91–1.04	0.394
Hyperlipidemia	0.97	0.91–1.04	0.420
History of alcohol intoxication	1.41	1.15–1.73	**0.001**
Malignancies	0.84	0.71–1.00	0.054
Heart failure	1.18	1.03–1.36	0.018
Atrial fibrillation	1.28	1.03–1.60	**0.027**
Smoking	1.16	0.43–3.09	0.769
Peripheral artery disease	1.09	0.91–1.31	0.331

p-Values less than 0.05 are presented as bold type

**Fig. 3:**
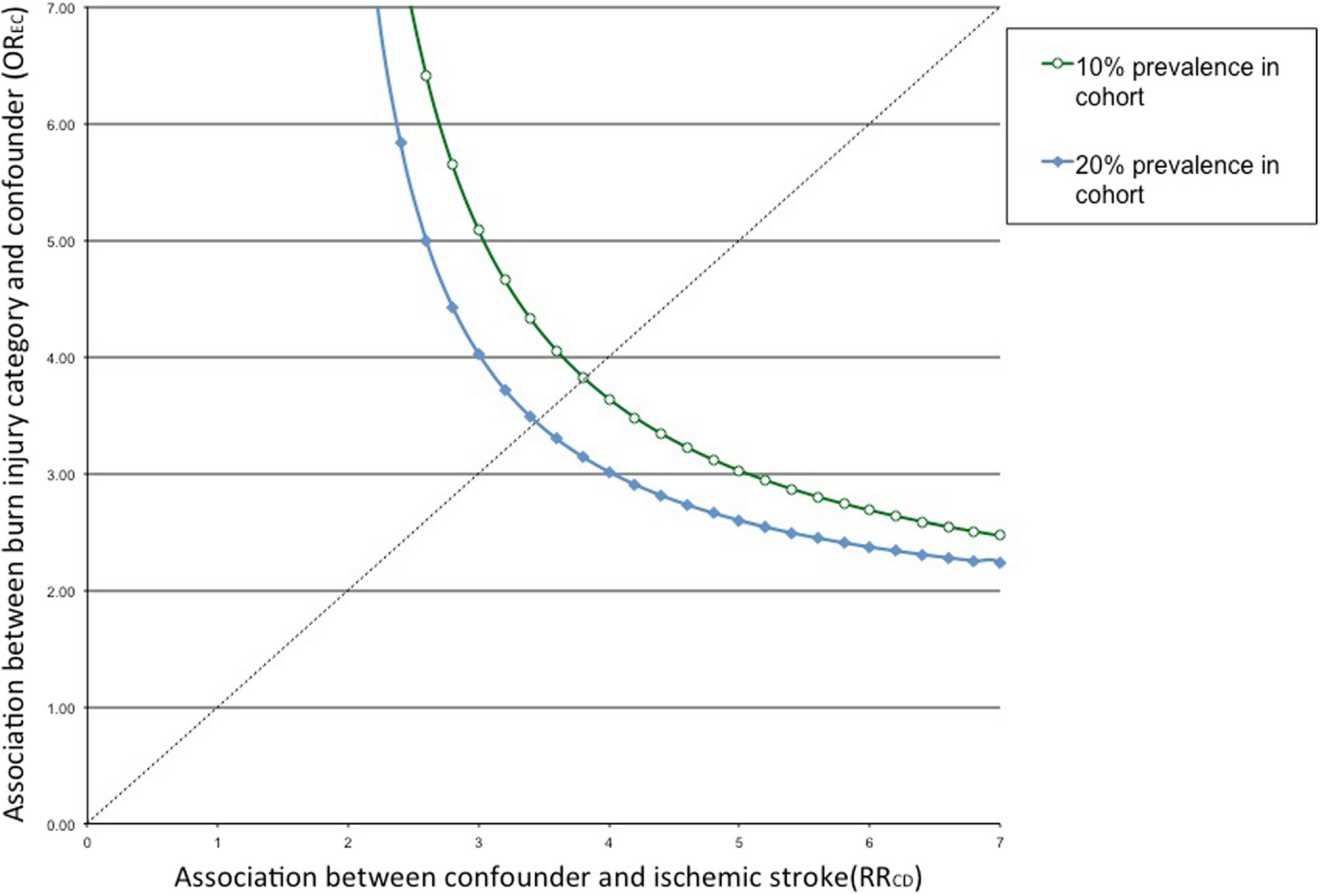
Sensitivity analyses of an unmeasured confounding

Sensitivity analyses showed that an unmeasured confounder present in 10 % of the matched cohort would be required to elevate the risk of ischemic stroke by a factor of 3.8 and would also be required to have a prevalence amongst burn injury patients of 3.8 times that observed amongst the unexposed group to explain a lower 95 % confidence limit HR of 1.43 (Fig. 3).

## Discussion

To our knowledge, this study represents the first systematic evaluation of the long-term risk between burn injury and ischemic stroke. We evaluated the risk of ischemic stroke onset in burn injury patients, utilizing the largest cohort to date. In our patient population, the risk of ischemic stroke was increased in patients with burn injury (HR 1.84; 95 % CI, 1.43–2.36) and still significant in those survived more than 12 months. (HR 1.54; 95 % CI, 1.11–2.13). These results reveal that burn injury patients have a higher risk of ischemic stroke that persists for more than one year post-injury. Burn injury prevention may therefore not only reduce the incidence of acute complications, but also ameliorate associated long-term morbidities.

Several hypotheses may be proposed to explain the higher risk of ischemic stroke in burn injury patients when compared to the general population. First, it is known that watershed stroke may occur in patients with compromised hemodynamic status [21, 22], and hypovolemia associated with the acute stage of burn injury patients may therefore account for the increased risk of stroke. Secondly, acute infection, a common complication in patients hospitalized with burn injury, may result in endothelial dysfunction, coagulopathy or direct platelet activation that may trigger or promote the cellular cascades associated with the onset of ischemic stroke [23, 24]. Finally, a hypercoagulable state known to occur during the post-burn recovery phase [25] may also elevate the risk for the development of stroke in patients with burn injury [26].

In our study, we evaluated the risk of ischemic stroke onset in burn injury patients, utilizing the largest cohort to date. The database was representative of the whole population of the country of Taiwan, and loss to follow-up or selection bias is, therefore, not a relevant concern. In a study by Cho et al. [10], stroke was reported to be a rare complication in patients admitted with burn injury. We advocate three possible reasons that could explain the discrepant results between their study and ours. First, the younger age of the patients in the Cho et al*.* [10] study than those comprising our study cohort may have been associated with reduced pre-existing vascular lesions such as atherosclerosis of brain vessels. Secondly, the patient population reported by Cho et al*.* [10] was enrolled from a single burn center, and the possibility exists that improved care (when compared to our patients from the general population) may have reduced the onset of medical complications. Finally, the severity of injury in patients evaluated by Cho et al., [10] may have been more severe, resulting in a reduced detection of stroke onset.

Another strength of the present study is that we extensively adjusted for possible confounding factors by applying hdPS analysis. In an administrative database, possible risk factors for ischemic stroke (such as functional status or dietary habits) cannot be assessed. However, a set of proxies may indirectly describe the overall status of patients. This status is viewed through the lenses of the health care system that provides the interventions [26]. A battery of proxies is a good overall proxy to account for the effect of unobserved confounding factors. The use of a large number of proxy covariates for propensity score estimation is known to effectively control for confounding factors in epidemiologic studies [22]. In our study, as many as 500 covariates were generated for analysis from the procedure codes, medication records and diagnostic codes recorded up to 2 years before entry of the follow-up data. In this way, we believe our study methods are more robust compared to traditional adjustment methods.

### Limitations

Our study was associated with several limitations. We used administrative data to generate our findings and ICD-9-CM codes for defining burn injury and ischemic stroke. These codes are often not as useful for precise operative definitions, since they are primarily used for insurance reimbursement. Thus, it is impossible to accurately determine the sensitivity, specificity, and clinical accuracy of the diagnoses when employing such data. To mitigate the number of false-positive cases, the inclusion criteria for burn injury and ischemic stroke patients were restricted to only those patients who had been hospitalized. The high PPVs that we observed validated the selection process, despite the size of our study and the use of a local database.

In the present study, the relationship between the affected body region(s) and risk of ischemic stroke in burn injury patients was not evaluated because we were able to obtain only limited information from the administrative database. Individual studies will be required to determine whether HRs differ significantly among different body regions (e.g., head versus torso).

In our study, subgroup analyses in patients with severe burn injuries to evaluate the severity-response effects were not performed. As mentioned previously, severe burn patients may have more comorbid risk factors (e.g., restricted mobility and higher mortality), possibly precluding ischemic stroke diagnosis.

Finally evaluation of other possible risk factors of burn injuries and ischemic stroke by the matched cohort method was complicated by the use of propensity score analyses to adjust for possible confounders. Although extensive adjustments for many possible covariates were made, the possibility of the presence of unmeasured confounders and overmatching bias remain. However, the adjusted HR for ischemic stroke in burn injury was sufficiently significant to indicate that the residual confounders may be unable to completely account for the results.

## Conclusion

The risk of ischemic stroke is significantly higher in patients hospitalized with burn injury than in the general population, and these risks may extend longer than expected.
